# Association between artificial intelligence literacy and empathic behavior among nursing interns: adjustment for moral distress and moral resilience

**DOI:** 10.3389/fmed.2026.1916897

**Published:** 2026-09-15

**Authors:** Chuanying Ding, Bingxia Zhang, Shijin Jia

**Affiliations:** Qilu Hospital of Shandong University (Qingdao), Qingdao, China

**Keywords:** artificial intelligence literacy, empathy, moral distress, moral resilience, nursing interns

## Abstract

**Background:**

Artificial intelligence (AI) literacy is increasingly relevant in nursing education, yet its association with empathic behavior among nursing interns remains unclear.

**Methods:**

This cross-sectional study included 300 nursing interns from three tertiary hospitals in China (December 2025–April 2026). AI literacy and empathic behavior were assessed using validated scales. Moral distress and moral resilience were included as covariates. Multivariable linear regression and restricted cubic spline analyses were performed, with subgroup analyses to assess robustness.

**Results:**

Higher AI literacy was significantly associated with greater empathic behavior in the unadjusted model (*β* = 4.40; 95% CI, 3.86–4.95; *P* < .001) and fully adjusted model (*β* = 4.31; 95% CI, 3.40–5.23; *P* < .001). No evidence of a nonlinear association was observed (P for nonlinearity = .886), and the association was broadly consistent across prespecified subgroups.

**Conclusions:**

Higher AI literacy was statistically associated with greater empathic behavior among nursing interns. Longitudinal and intervention studies are needed to determine the temporal and causal relationships between these constructs.

## 1 Introduction

Artificial intelligence (AI) technologies are rapidly transforming contemporary healthcare systems and nursing education environments (1, 2). Recent advances in generative AI, intelligent clinical documentation systems, decision-support algorithms, and conversational AI tools have substantially changed how healthcare information is accessed, interpreted, and communicated in clinical settings (3–5). Nursing students and interns are increasingly exposed to AI-assisted technologies during clinical training, patient education, and information retrieval processes (6). Consequently, AI-related competencies are becoming an important component of modern nursing professionalism and clinical preparedness (7, 8). Recent literature has emphasized that future healthcare professionals are expected not only to use AI technologies efficiently, but also to critically evaluate AI-generated information and integrate digital technologies into patient-centered care delivery (9).

Despite these technological advances, concerns have emerged regarding the potential influence of AI on humanistic care and interpersonal interactions in healthcare (10). Empathy represents one of the core professional competencies in nursing practice and is widely recognized as essential for effective therapeutic communication, patient trust, clinical satisfaction, and quality of care (11). Empathic behavior refers to the ability to understand patients' emotional experiences and respond with appropriate emotional and behavioral support (12, 13). Previous studies have demonstrated that higher empathy among nurses and nursing students is associated with improved patient outcomes, lower levels of burnout, stronger professional identity, and better clinical communication performance (14–16). However, empathy may be vulnerable to increasing technological complexity, emotional exhaustion, ethical conflicts, and cognitive overload within modern clinical environments. Clinical internship is a particularly critical period for the development of empathic behavior. During this transitional stage, nursing interns are required to adapt simultaneously to clinical responsibilities, professional identity formation, interpersonal communication demands, and emotionally challenging care situations (17, 18). Existing evidence suggests that nursing interns frequently experience stress related to workload, uncertainty in clinical decision-making, role ambiguity, and ethical dilemmas, all of which may negatively influence empathic engagement with patients (19). Maintaining empathic behavior under such conditions may therefore depend not only on emotional capacity but also on the availability of cognitive and psychological resources that support adaptive functioning in complex clinical environments.

Within increasingly digitalized healthcare systems, AI literacy has emerged as an important competency that may influence healthcare professionals' adaptive functioning. AI literacy generally refers to the ability to understand, critically evaluate, and appropriately interact with AI-generated information and systems. Contemporary frameworks conceptualize AI literacy as a multidimensional competency encompassing navigation skills, evaluative judgment, and understanding of AI mechanisms (20). Importantly, AI literacy extends beyond technical proficiency and includes the capacity to critically appraise AI outputs, recognize their limitations, and retain appropriate professional judgment when interacting with AI-supported systems. Recent studies have suggested that insufficient AI literacy may contribute to uncertainty, information overload, reduced confidence, and impaired communication in healthcare environments (21–23). Conversely, higher AI literacy may provide cognitive and professional resources for navigating complex information environments, supporting critical judgment and adaptive clinical functioning (24, 25)_._

The potential relevance of AI literacy to empathic behavior can be further understood through Conservation of Resources theory (26, 27). COR theory proposes that individuals strive to acquire, retain, and protect valued resources, and that access to adequate personal and contextual resources may support adaptive functioning under demanding circumstances (26). From this perspective, AI literacy may be conceptualized as a cognitive and professional resource that helps nursing interns manage AI-mediated information, evaluate potentially uncertain outputs, and navigate technology-intensive clinical environments while maintaining professional judgment. Such resource availability may be associated with greater capacity to remain attentive and responsive to patients' emotional and interpersonal needs. However, this theoretical perspective does not imply that AI literacy directly causes greater empathy; rather, it provides a plausible conceptual basis for examining whether these constructs are associated in nursing interns.

The relationship between AI literacy and empathic behavior may also be considered within the broader context of ethical adaptation during clinical internship. Moral distress, defined as the psychological disequilibrium experienced when individuals are unable to act according to their ethical beliefs because of internal or external constraints, has become an important concern among nurses and nursing trainees (25). Persistent moral distress represents a form of psychological strain and resource loss that may compromise emotional well-being and patient-centered care. In contrast, moral resilience refers to the capacity to sustain or restore integrity and ethical functioning in morally challenging situations and may represent a resource that supports adaptive responses to ethical adversity. Emerging evidence suggests that moral distress is associated with adverse psychological and professional outcomes, whereas moral resilience may help healthcare professionals maintain ethical functioning and compassionate care in challenging clinical contexts. Accordingly, moral distress and moral resilience may be relevant factors when examining the association between AI literacy and empathic behavior, because differences in ethical stress and resilience may independently influence nurses' capacity for empathic engagement. Rather than treating these variables as established mediating mechanisms, the present study therefore considered them as important ethical-psychological factors for statistical adjustment.

Despite growing research on AI competencies in healthcare education, existing studies have predominantly examined AI acceptance, attitudes, anxiety, readiness, or educational preparedness among nursing students and healthcare professionals (21–24). Direct empirical evidence regarding whether AI literacy is associated with empathic behavior among nursing interns remains limited. Moreover, the extent to which this association persists after accounting for ethical-psychological factors such as moral distress and moral resilience has not been adequately examined. This gap is particularly relevant during clinical internship, when nursing students simultaneously develop professional competencies, adapt to technology-intensive care environments, and encounter ethically challenging patient-care situations. Therefore, the scientific question of the present study was whether AI literacy is associated with empathic behavior among nursing interns and whether this association remains after adjustment for moral distress and moral resilience. The present study aimed to examine the association between AI literacy and empathic behavior among nursing interns and to determine whether the observed association remained independent after adjustment for moral distress and moral resilience. We hypothesized that higher AI literacy would be positively associated with greater empathic behavior among nursing interns. Given the cross-sectional design, the hypothesis concerns an association rather than a causal effect. By clarifying the relationship between AI-related competency and empathic behavior while accounting for relevant ethical-psychological factors, this study may contribute to a more integrated understanding of how nursing interns adapt to increasingly AI-enabled clinical environments and may inform nursing education that seeks to balance technological competence with humanistic care.

## 2 Methods

### 2.1 Study design and participants

This cross-sectional study was conducted among nursing interns from three tertiary hospitals in China between December 2025 and April 2026. Convenience sampling was used to recruit nursing interns from the participating hospitals. Nursing interns were defined as nursing students who had completed their classroom-based nursing education and were undertaking supervised clinical internship training in hospital settings as part of their pre-licensure nursing education. Eligible participants were aged 18 years or older and were undertaking clinical practice at one of the participating hospitals during the study period. Participants were excluded if they had a history of major psychiatric or psychological trauma or were not participating in clinical rotations because of sick leave, personal leave, or other prolonged absence. Eligible nursing interns were identified from the clinical internship rosters maintained by the nursing education departments of the participating hospitals.

An *a priori* sample size calculation was performed using G*Power version 3.1 for multiple linear regression. Because no reliable effect-size estimates were available for the association between AI literacy and empathic behavior among nursing interns, a medium effect size (f^2^ = 0.15), a 2-sided *α* level of.05, and 80% statistical power were assumed based on Cohen's recommendations. Under these assumptions, the minimum required sample size was 218 participants. A total of 318 eligible nursing interns were invited to participate. Of these, 300 completed the questionnaire and were included in the final analysis, yielding a response rate of 94.3%. Participants received no financial or material compensation for completing the survey.

This study was approved by the Institutional Ethics Committee of Qilu Hospital of Shandong University (Qingdao) (approval No. KYLL-2026052) before participant recruitment. All study procedures were conducted in accordance with the Declaration of Helsinki and relevant institutional guidelines. This study was conducted and reported in accordance with the Strengthening the Reporting of Observational Studies in Epidemiology (STROBE) guideline (Supplementary File 1).

### 2.2 Measures

#### 2.2.1 Sociodemographic characteristics

Sociodemographic information was collected using a structured questionnaire, including sex (male or female), age, educational level (junior college, undergraduate, or postgraduate), only-child status (yes or no), internship stage (early stage [≤3 months], middle stage [4–8 months], or late stage [≥9 months]), reasons for choosing the nursing profession (self-choice, family recommendation, admission adjustment, or other), and future career planning (committed to nursing work, possible career change, or uncertain).

#### 2.2.2 AI literacy

AI literacy was assessed using the 12-item AI literacy scale adapted by Reinhardt et al. from the Social Media Information Literacy (SMIL) framework originally developed by Heiss et al. (20, 28). The adapted instrument was developed to assess AI literacy in the context of generative AI systems rather than social media information literacy (20, 28). This instrument conceptualizes AI literacy as a multidimensional competency reflecting individuals' ability to navigate, critically evaluate, and understand AI-based systems. It comprises 3 domains—navigation, appraisal, and comprehension—with 4 items in each domain. Navigation assesses the ability to effectively interact with generative AI systems to obtain relevant information; appraisal evaluates the ability to critically assess the accuracy and credibility of AI-generated content; and comprehension assesses understanding of how generative AI systems generate responses. Because the adapted instrument had not been comprehensively validated specifically among nursing interns, a standardized forward–backward translation procedure was undertaken for the Chinese version used in this study. Two independent bilingual translators independently performed forward translations, which were reconciled into a single Chinese version through discussion within the research team. A third bilingual translator who was blinded to the original instrument performed the back-translation. The pre-final Chinese version was pilot-tested among 30 nursing interns who were not included in the main study, and minor wording modifications were made based on participants' feedback regarding clarity and comprehensibility. All items are rated on a 7-point Likert scale ranging from 1 (very difficult) to 7 (very easy). The overall AI literacy score is calculated as the mean of the 12 item scores, yielding a possible score range of 1 to 7, with higher scores indicating greater AI literacy. In the present study, the scale demonstrated excellent internal consistency (Cronbach's *α* = .975), with Cronbach's *α* coefficients of.927,.932, and.927 for the navigation, appraisal, and comprehension domains, respectively. Exploratory factor analysis supported the expected 3-factor structure (Kaiser–Meyer–Olkin measure >0.70; Bartlett's test of sphericity, *P* < .001).

#### 2.2.3 Empathic behavior

Empathic behavior was assessed using the Chinese version of the Jefferson Scale of Empathy–Health Professionals (JSE-HP). The original scale was developed by Hojat et al. to evaluate empathy among healthcare professionals and was subsequently translated and culturally adapted into Chinese (29). The scale comprises 20 items across 3 dimensions: perspective taking, compassionate care, and standing in the patient's shoes. Responses are rated on a 7-point Likert scale ranging from 1 (“strongly disagree”) to 7 (“strongly agree”). Total scores are obtained by summing all item scores, with higher scores indicating greater empathic behavior. In the present study, the JSE-HP demonstrated acceptable-to-excellent internal consistency (Cronbach's *α* = 0.874).

#### 2.2.4 Moral distress

Moral distress was measured using the Chinese version of the Moral Distress Scale–Revised (MDS-R), originally developed by Corley et al. for healthcare professionals (30). The Chinese version was translated and culturally adapted and has demonstrated acceptable reliability and validity among Chinese nursing populations. The instrument contains 22 items across 4 dimensions: individual responsibility, inability to protect patients' best interests, value conflict, and patient harm. Each item evaluates both the frequency and intensity of morally distressing situations using a 5-point Likert scale ranging from 0 to 4. Item scores are calculated as the product of frequency and intensity scores, and the total score ranges from 0 to 352, with higher scores indicating greater moral distress. In the present study, the MDS-R demonstrated a Cronbach's *α* of 0.855.

#### 2.2.5 Moral resilience

Moral resilience was assessed using the Chinese version of the Rushton Moral Resilience Scale (RMRS). The original RMRS was developed by Heinze et al. to evaluate healthcare professionals' capacity to maintain or restore integrity when confronting ethical adversity and moral complexity (31). The Chinese version has undergone translation and cultural adaptation for Chinese healthcare populations and has demonstrated satisfactory reliability and validity. The scale consists of 16 items across 4 domains: responses to moral adversity, personal integrity, relational integrity, and moral efficacy. Each item is rated on a 4-point Likert scale ranging from 1 (“disagree”) to 4 (“agree”), with negatively worded items reverse scored. The total score is obtained by summing all 16 items and ranges from 16 to 64, with higher scores indicating greater moral resilience. In the present study, the RMRS-16 demonstrated a Cronbach's *α* of 0.912.

#### 2.2.6 Covariates

Potential covariates included age, sex, educational level, only-child status, internship stage, reasons for choosing the nursing profession, future career planning, moral distress, and moral resilience. These variables were prespecified based on previous literature and their conceptual relevance to empathic behavior and the clinical and ethical context of nursing internship (32, 33). Covariate selection was intended to account for potential confounding rather than to identify variables that were statistically significant predictors of empathic behavior in univariable analyses. Accordingly, candidate covariates were not selected solely on the basis of their statistical significance in univariable analyses, because reliance on bivariable or univariable significance testing may exclude important confounders and potentially bias estimates of the association of interest.

### 2.3 Data collection and quality control

Data collection was conducted using the Wenjuanxing online survey platform. The questionnaires were distributed directly to eligible nursing interns by members of the research team at the participating hospitals. Prior to completing the survey, participants were informed of the study purpose and procedures and provided electronic informed consent.

To improve data quality and completeness, all questionnaire items were configured as mandatory within the survey system. After data collection, questionnaires were reviewed for completeness, internal consistency, and logical accuracy before inclusion in the final dataset. Records with missing information on key exposure or outcome variables were excluded from the analysis.

### 2.4 Statistical analysis

All statistical analyses were performed using R version 4.2.1. Continuous variables are presented as means and standard deviations (SDs), whereas categorical variables are presented as frequencies and percentages.

Linear regression models were used to examine the associations between AI literacy and empathic behavior. Three models were constructed. Model 1 was an unadjusted model including AI literacy only. Model 2 was adjusted for age, sex, educational level, only-child status, internship stage, reasons for choosing the nursing profession, and future career planning. Model 3 was additionally adjusted for moral distress and moral resilience to further evaluate the independent association between AI literacy and empathic behavior.

Restricted cubic spline analyses with 3 knots were further performed to explore potential nonlinear associations between AI literacy and empathic behavior. Subgroup analyses were conducted according to sex, educational level, only-child status, internship stage, reasons for choosing the nursing profession, and future career planning to evaluate the consistency of associations across participant characteristics. Regression coefficients (*β*s) and corresponding 95% confidence intervals (CIs) were reported. A 2-sided *P* value <.05 was considered statistically significant.

## 3 Results

### 3.1 Participant characteristics

A total of 300 nursing interns were included in the final analysis. The mean (SD) age of the participants was 22.0 (1.2) years. Among them, 234 participants (78.0%) were female and 66 (22.0%) were male. Most participants were pursuing a bachelor's degree [176 (58.7%)], were not only children [185 (61.7%)], and reported a definite intention to pursue a nursing career in the future [178 (59.3%)]. Regarding internship stage, 125 participants (41.7%) were in the middle stage of internship, followed by 89 (29.7%) in the late stage and 86 (28.7%) in the early stage.

The mean (SD) moral distress score was 142.2 (28.7), the mean (SD) moral resilience score was 43.8 (5.8), and the mean (SD) empathy score was 105.4 (8.3) (Table 1).

**Table 1:** Baseline characteristics of nursing interns.

Characteristics	Number	%	Mean	SD
Age, y	—	—	22	1.2
Sex				
Male	66	22	—	—
Female	234	78	—	—
Educational level				
Junior college	98	32.7	—	—
Bachelor's degree	176	58.7	—	—
Master's degree	26	8.7	—	—
Only-child status				
Yes	115	38.3	—	—
No	185	61.7	—	—
Internship stage				
Early stage (≤3 months)	86	28.7	—	—
Middle stage (4–8 months)	125	41.7	—	—
Late stage (≥9 months)	89	29.7	—	—
Motivation for choosing nursing				
Self-motivated choice	144	48	—	—
Family recommendation	77	25.7	—	—
Admission adjustment	49	16.3	—	—
Other reasons	30	10	—	—
Future career plan				
Definitely pursue nursing career	178	59.3	—	—
May change profession	64	21.3	—	—
Uncertain	58	19.3	—	—
Moral distress score	—	—	142.2	28.7
Moral resilience score	—	—	43.8	5.8
Empathy score	—	—	105.4	8.3

### 3.2 Association between AI literacy and empathy

Linear regression analyses demonstrated a significant positive association between AI literacy and empathy among nursing interns (Table 2). In the unadjusted model (Model 1), higher AI literacy was significantly associated with higher empathy scores (*β* = 4.40; 95% CI, 3.86 to 4.95; *P* < .001). The association remained statistically significant after adjustment for sociodemographic and internship-related characteristics in Model 2 (*β* = 4.46; 95% CI, 3.91 to 5.02; *P* < .001).

**Table 2:** Association between AI literacy and empathy among nursing interns.

Variables	Model 1 *β* (95% CI)	*P* value	Model 2 β (95% CI)	*P* value	Model 3 β (95% CI)	*P* value
AI literacy	4.40 (3.86 to 4.95)	<0.001	4.46 (3.91 to 5.02)	<0.001	4.31 (3.40 to 5.23)	<0.001
Age (years)	—	—	−0.37 (−0.95 to 0.21)	0.210	−0.36 (−0.94 to 0.22)	0.221
Sex (ref: male)						
Female	—	—	0.48 (−1.23 to 2.20)	0.579	0.42 (−1.31 to 2.15)	0.633
Education level (ref: junior college)						
Bachelor's degree	—	—	−0.01 (−1.56 to 1.54)	0.990	−0.04 (−1.59 to 1.51)	0.960
Master's degree	—	—	−0.54 (−3.23 to 2.16)	0.696	−0.57 (−3.27 to 2.13)	0.678
Only child status (ref: no)						
Yes	—	—	1.10 (−0.35 to 2.55)	0.135	1.15 (−0.32 to 2.61)	0.125
Internship stage (ref: early stage)						
Middle stage	—	—	0.28 (−1.44 to 2.00)	0.750	0.26 (−1.48 to 1.99)	0.770
Late stage	—	—	0.43 (−1.42 to 2.28)	0.648	0.35 (−1.53 to 2.22)	0.718
Motivation for choosing nursing (ref: self-motivated choice)						
Family recommendation	—	—	−0.30 (−2.01 to 1.41)	0.731	−0.35 (−2.07 to 1.38)	0.692
Admission adjustment	—	—	−0.68 (−2.68 to 1.32)	0.503	−0.69 (−2.70 to 1.32)	0.500
Other reasons	—	—	1.55 (−0.92 to 4.02)	0.217	1.49 (−0.98 to 3.97)	0.236
Future career plan (ref: definitely pursue nursing career)						
May change profession	—	—	−0.42 (−2.21 to 1.36)	0.641	−0.47 (−2.28 to 1.35)	0.615
Uncertain	—	—	−2.86 (−4.72 to −1.00)	0.003	−2.86 (−4.72 to −1.00)	0.003
Moral distress score	—	—	—	—	0.01 (−0.03 to 0.04)	0.734
Moral resilience score	—	—	—	—	0.07 (−0.11 to 0.25)	0.456

Model 1: Unadjusted model.

Model 2: Adjusted for age, sex, education level, only-child status, internship stage, motivation for choosing nursing, and future career planning.

Model 3: Further adjusted for moral distress and moral resilience scores.

Further adjustment for moral distress and moral resilience in Model 3 did not materially alter the association (*β* = 4.31; 95% CI, 3.40 to 5.23; *P* < .001), suggesting that the relationship between AI literacy and empathy was independent of ethical distress and adaptive resilience factors.

Among the covariates included in the fully adjusted model, participants who reported uncertainty regarding future career planning demonstrated significantly lower empathy scores compared with those who intended to definitely pursue a nursing career (*β* = −2.86; 95% CI, −4.72 to −1.00; *P* = .003). No statistically significant associations were observed for age, sex, educational level, only-child status, internship stage, motivation for choosing nursing, moral distress, or moral resilience (all *P* > .05) (Table 2).

### 3.3 Nonlinear association between AI literacy and empathy

Restricted cubic spline analyses were performed to further examine the potential nonlinear association between AI literacy and empathy (Figure 1). The overall association between AI literacy and empathy was statistically significant (*P* overall <.0001). However, no evidence of a nonlinear relationship was observed (P for nonlinearity = .886), indicating that the association between AI literacy and empathy was approximately linear across the observed range of AI literacy scores.

**Figure 1:**
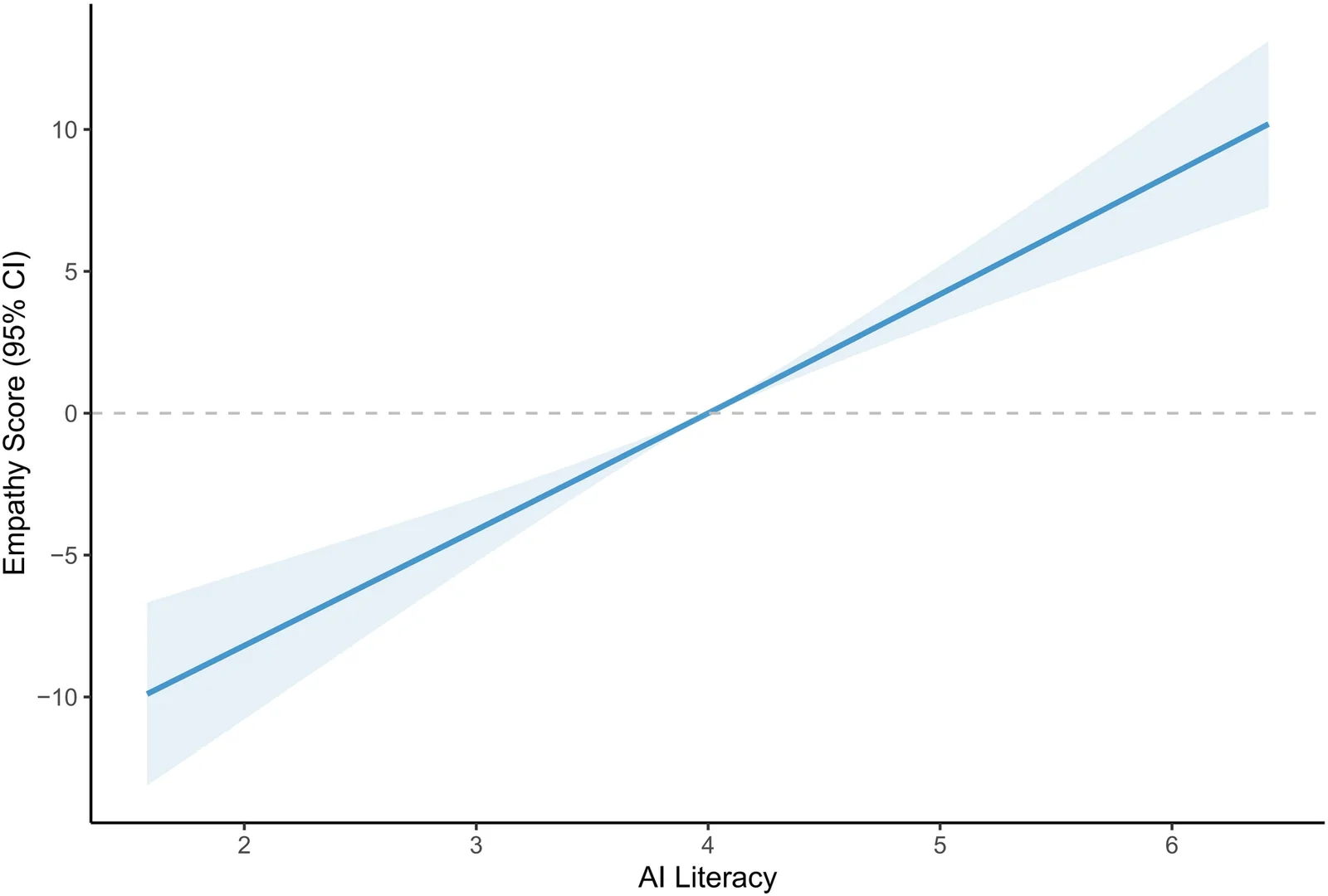
Nonlinear association between AI literacy and empathy.

### 3.4 Subgroup analyses

Subgroup analyses were conducted to assess the consistency of the association between AI literacy and empathy across participant characteristics (Figure 2). Overall, higher AI literacy was consistently associated with higher empathy scores across most subgroups.

**Figure 2:**
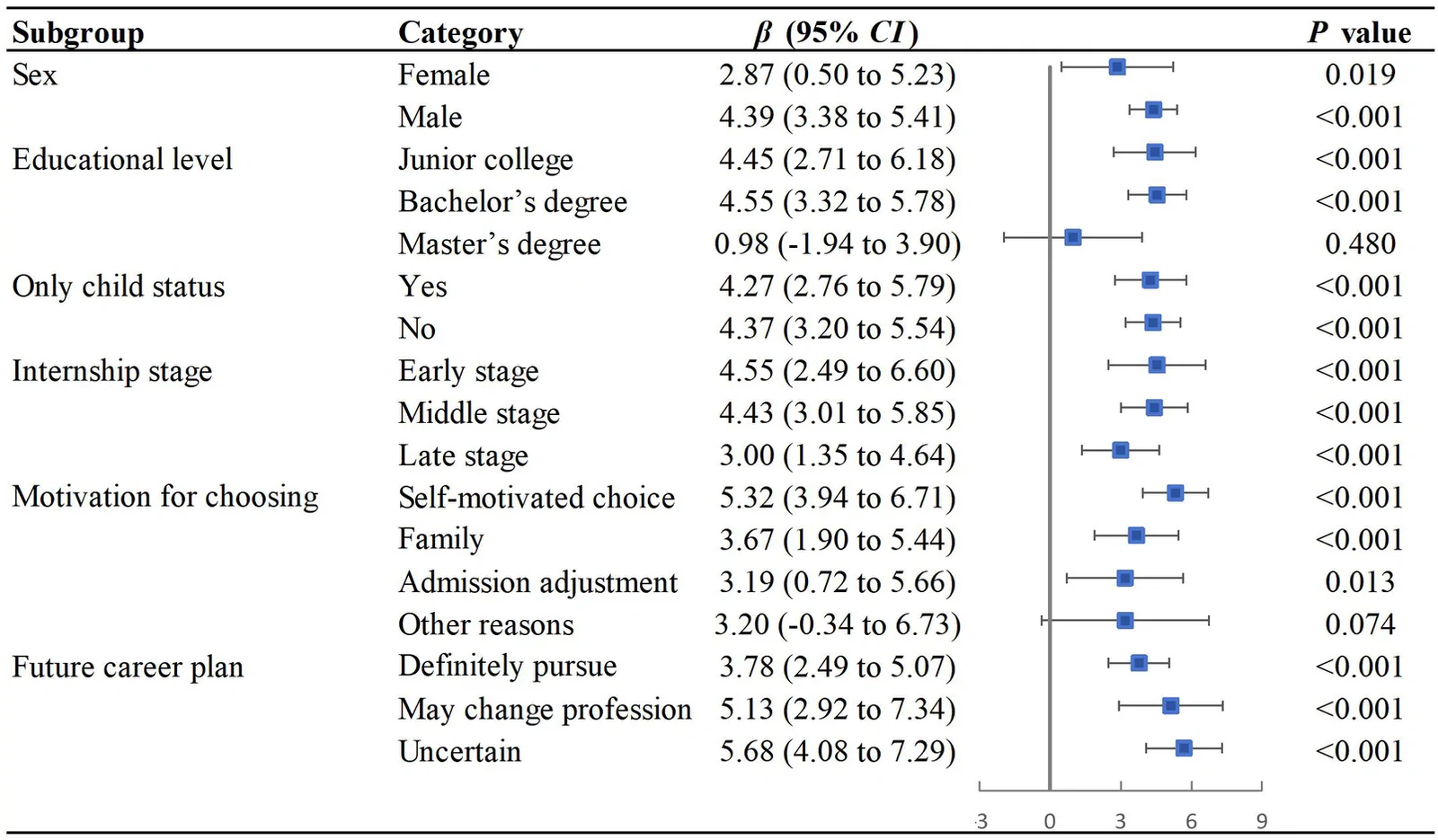
Subgroup analyses of the association between AI literacy and empathy among nursing interns.

Positive associations were observed in both female (*β* = 2.87; 95% CI, 0.50 to 5.23) and male participants (*β* = 4.39; 95% CI, 3.38 to 5.41). Similar associations were identified across educational levels, only-child status categories, internship stages, motivations for choosing nursing, and future career planning groups.

Across educational backgrounds, the association was strongest among participants with junior college education (*β* = 4.45; 95% CI, 2.71 to 6.18) and bachelor's degree education (*β* = 4.55; 95% CI, 3.32 to 5.78), whereas the association did not reach statistical significance among postgraduate participants (*β* = 0.98; 95% CI, −1.94 to 3.90).

Across internship stages, the positive association between AI literacy and empathy remained stable, with *β* coefficients ranging from 3.00 to 4.55. Participants who selected nursing through self-motivated choice demonstrated the strongest association (*β* = 5.32; 95% CI, 3.94 to 6.71). Similarly, stronger positive associations were observed among participants who were uncertain about future career plans (*β* = 5.68; 95% CI, 4.08 to 7.29) and those considering a career change (*β* = 5.13; 95% CI, 2.92 to 7.34).

## 4 Discussion

In this cross-sectional study of nursing interns, higher AI literacy was consistently associated with greater empathy, and the association remained robust after adjustment for demographic characteristics, internship-related factors, moral distress, and moral resilience. Restricted cubic spline analyses further demonstrated no evidence of a nonlinear association across the observed range of AI literacy scores. In addition, subgroup analyses suggested that the association was generally consistent across sex, educational background, internship stage, and professional planning categories. Taken together, these findings indicate a statistical association between AI literacy and empathy in this sample. Consistent with the theoretical framework introduced above, AI literacy may be conceptualized as a cognitive and professional resource that is associated with adaptive functioning in technology-intensive clinical environments; however, the present findings do not establish that AI literacy itself produces higher empathy.

Empathy is widely recognized as a foundational component of high-quality nursing care and patient-centered practice. Previous research has demonstrated that empathy is associated with improved nurse–patient communication, greater patient satisfaction, enhanced therapeutic alliance, and better psychological outcomes among patients, while also contributing to professional identity formation and occupational meaning among nurses (34–36). However, maintaining empathic engagement during clinical training can be challenging. Nursing interns frequently experience emotional exhaustion, workload burden, ethical uncertainty, and role-transition stress, all of which may impair empathic responsiveness (37, 38). Against this background, the present findings extend the existing literature by identifying a positive statistical association between AI literacy and empathy among nursing interns. However, the cross-sectional nature of the data precludes determining whether higher AI literacy precedes greater empathy or whether the two characteristics develop concurrently or influence each other over time.

The positive association between AI literacy and empathy may appear counterintuitive because technological advancement is often assumed to reduce interpersonal interaction and humanistic care (39). Existing concerns surrounding AI in healthcare frequently emphasize depersonalization, automation bias, and diminished emotional connection in clinical practice (3, 40, 41). Nevertheless, emerging evidence increasingly suggests that technological competency and humanistic competency are not inherently contradictory (42, 43). One possible interpretation is that individuals who are more comfortable navigating and critically evaluating digital technologies may also possess broader cognitive or professional characteristics that are relevant to interpersonal functioning. However, because these characteristics were not experimentally manipulated or assessed longitudinally in the present study, this interpretation should be regarded as a plausible explanation rather than an established mechanism (44–46).

Several potential mechanisms may explain the observed association. First, AI literacy may be associated with how nursing interns perceive and manage information complexity (45). Nursing interns often encounter large volumes of fragmented information, rapidly evolving clinical demands, and limited decisional autonomy. Individuals with higher AI literacy may be better equipped to interpret, evaluate, and contextualize AI-generated or digitally mediated information, which could coexist with lower perceived informational uncertainty or cognitive burden. According to cognitive load theory, reduced cognitive burden may preserve attentional and emotional resources necessary for empathic engagement (47). This interpretation remains hypothetical, because cognitive load and informational uncertainty were not directly measured in the present study. Second, AI literacy may share conceptual features with broader metacognitive and reflective competencies that are also relevant to empathy. Contemporary conceptualizations of AI literacy increasingly emphasize not only operational skills, but also critical thinking, reflective judgment, ethical awareness, and contextual interpretation (23). These higher-order cognitive abilities overlap conceptually with components of clinical empathy, including perspective-taking, situational understanding, and reflective responsiveness (48). Nursing interns with stronger AI literacy may therefore demonstrate greater openness to multiple perspectives and more adaptive interpersonal processing during patient interactions (49). Third, AI literacy may be correlated with professional self-efficacy and adaptive confidence in digitally evolving healthcare environments. Self-efficacy theory proposes that individuals who perceive greater competence in managing environmental demands are more likely to demonstrate constructive coping behaviors and positive interpersonal functioning (50). In clinical practice, nursing interns who possess stronger confidence in understanding and evaluating AI-assisted information may experience less anxiety regarding technological uncertainty and role inadequacy. Reduced professional insecurity may subsequently facilitate more authentic and emotionally available patient interactions.

An important aspect of the present findings is that the association between AI literacy and empathy remained statistically significant even after adjustment for moral distress and moral resilience. This finding suggests that the observed association was not fully accounted for by the measured levels of these two ethical-psychological factors in the adjusted models. However, it should not be interpreted as evidence that moral distress or moral resilience are unrelated to the association. Moral distress and moral resilience may operate through other pathways, interact with other individual or contextual characteristics, or play different roles under specific clinical circumstances (51, 52). Moreover, statistical adjustment cannot determine whether these variables function as confounders, mediators, moderators, or correlates without longitudinal or experimental evidence. Accordingly, the present results should be interpreted as demonstrating an association between AI literacy and empathy that persisted after accounting for measured moral distress and moral resilience, rather than as establishing an effect independent of these constructs.

The subgroup analyses further strengthened the robustness of the overall findings. Positive associations between AI literacy and empathy were observed across most demographic and professional subgroups, suggesting that the relationship was relatively stable across different educational and experiential contexts. Notably, the association appeared somewhat attenuated among postgraduate participants and individuals who selected nursing for “other reasons,” although these subgroup estimates should be interpreted cautiously because of relatively limited sample sizes. These differences may reflect variation in educational trajectory, professional motivation, learning orientation, or other unmeasured characteristics; however, the present study was not designed or powered to establish whether these factors modify the association. Future longitudinal research could examine whether professional identity, vocational commitment, or learning orientation are associated with differences in the relationship between AI literacy and interpersonal competencies. The finding that the association between AI literacy and empathy was approximately linear also has important implications. The absence of a nonlinear threshold effect suggests that we did not identify a clear threshold or nonlinear pattern in the observed association across the measured range of AI literacy scores. This finding does not establish that incremental increases in AI literacy would produce corresponding increases in empathy. Rather, it indicates that the statistical relationship observed in this sample was reasonably compatible with a linear form. Future longitudinal and intervention studies are needed to determine whether changes in AI literacy are accompanied by changes in empathy and whether any clinically meaningful threshold exists.

Current discussions surrounding AI in healthcare frequently focus on efficiency, automation, and clinical decision support, whereas considerably less attention has been devoted to how AI competencies intersect with humanistic dimensions of care (53, 54). Yet empathy remains one of the most irreplaceable aspects of nursing practice. As AI systems become increasingly involved in documentation, information retrieval, triage support, and patient monitoring, the role of nurses may shift toward more relational, interpretive, and emotionally responsive functions (55). In this context, cultivating AI literacy may paradoxically become increasingly important for preserving human-centered care rather than diminishing it. From a practical standpoint, the findings suggest that nursing education programs should move beyond viewing AI training solely as technical instruction. Instead, AI literacy education may need to be embedded within broader frameworks of clinical reasoning, ethical reflection, communication training, and patient-centered care. Educational programs that integrate critical evaluation of AI-generated information, algorithmic bias awareness, reflective decision-making, and human-machine collaboration may help prepare future nurses for increasingly complex sociotechnical clinical environments. Importantly, such educational strategies should emphasize that AI is intended to support rather than replace empathic nursing care.

### 4.1 Limitations

Several limitations should be acknowledged. First, the cross-sectional design precludes causal inference, and longitudinal studies are required to clarify the temporal relationship between AI literacy and empathy. Second, all variables were measured using self-reported instruments, which may introduce reporting bias and social desirability effects. Third, participants were recruited from tertiary hospitals in China, potentially limiting the generalizability of the findings to other healthcare systems, educational contexts, or cultural settings. Fourth, although multiple covariates were adjusted for, residual confounding from unmeasured factors such as personality traits, emotional intelligence, burnout, or prior digital technology exposure cannot be excluded. Finally, empathy is a multidimensional construct, and future studies incorporating behavioral assessments or patient-reported evaluations may provide a more comprehensive understanding of empathic practice.

## 5 Conclusion

The present study contributes to the emerging literature examining the relationship between digital competency and humanistic care in healthcare education. Among nursing interns, higher AI literacy was statistically associated with greater empathy after adjustment for measured demographic, internship-related, moral distress, and moral resilience variables. These findings provide a basis for further investigation of the relationship between AI-related competencies and humanistic dimensions of nursing practice, but they do not establish a causal effect of AI literacy on empathy. As healthcare systems continue to evolve toward AI-supported models of care, longitudinal and intervention studies are warranted to clarify the temporal direction of this association, evaluate potential reverse causality and underlying mechanisms, and determine whether AI literacy education is associated with changes in empathic behavior.

## Data Availability

The raw data supporting the conclusions of this article will be made available by the authors, without undue reservation.
